# A decade of progress: a before and after study on improved survival in pancreatic cancer through evolving multimodal therapy

**DOI:** 10.1097/JS9.0000000000005024

**Published:** 2026-03-17

**Authors:** Ting-Kai Liao, Chih-Jung Wang, Wei-Hsun Lu, Ping-Jui Su, Chien-Jui Huang, Yung-Yeh Su, Chih-Chieh Yen, I-Ting Liu, Chia-Jui Yen, Ying Jui Chao, Yan-Shen Shan

**Affiliations:** a Department of Surgery, National Cheng Kung University Hospital, College of Medicine, National Cheng Kung University, Tainan, Taiwan; b Institute of Clinical Medicine, College of Medicine, National Cheng Kung University, Tainan, Taiwan; c Department of Internal Medicine, National Cheng Kung University Hospital, College of Medicine, National Cheng Kung University, Tainan, Taiwan; d Department of Oncology, National Cheng Kung University Hospital, College of Medicine, National Cheng Kung University, Tainan, Taiwan; e National Institute of Cancer Research, National Health Research Institute, Tainan, Taiwan

**Keywords:** before and after study, multimodal therapy, pancreatic cancer, pancreatic surgery, survival outcomes

## Abstract

**Background:**

Pancreatic cancer remains one of the deadliest malignancies with poor survival outcomes. This study investigated the influence of evolving multimodal strategies, particularly the increased use of neoadjuvant chemotherapy (NAC) and extended surgical resection, on patient outcomes over the last decade.

**Methods:**

This before-and-after study included patients with pancreatic cancer who received curative surgery at a tertiary medical center from 2011 to 2022. The patients were divided into two treatment periods: Period A (2011–2017, *n* = 154) and Period B (2018–2022, *n* = 251). Patients were evaluated based on their clinical stage, resectability, treatment strategy, surgical approach, and survival outcomes. The primary endpoints were overall survival (OS) and progression-free survival (PFS). Sensitivity analysis was conducted by propensity score matching to minimize the differences in clinical demographics.

**Results:**

NAC use increased significantly from 22.1% in period A to 74.9% in period B (*P* < 0.001), along with an increase in the use of extended pancreatectomies (31%–58%, *P* < 0.001). Extended resections provided survival benefits yet were associated with higher major morbidity (20% vs 11%) and 30-day mortality rates (2.1% vs 0.5%). Median OS and PFS improved markedly from A to B for resectable/borderline resectable cases (OS: 19.2–35.5 months; PFS: 8.3–18.6 months; both *P* < 0.001). Improvements in OS from period A to period B were also observed for patients with locally advanced/metastatic disease (12.3–19.7 months, *P* < 0.001). Multivariate analysis identified period B, lower CA19-9 levels before surgery, early pathological stage, and adjuvant chemotherapy as independent predictors of survival. Similar results were determined after propensity score matching.

**Conclusion:**

The shift toward a multimodal approach significantly improved survival in patients with pancreatic cancer, especially those with resectable and borderline resectable tumors. Although extended pancreatectomy increases surgical risk, the survival benefits support its selective use. Continued refinement of the treatment selection and perioperative care is warranted.

## Introduction

Pancreatic ductal adenocarcinoma (PDAC) is a formidable clinical challenge and ranks among the leading causes of cancer-related mortality worldwide^[^1,2^]^. Historically, the prognosis of PDAC has been dismal, primarily because of late diagnosis, limited response to systemic therapies, and surgical difficulties with locally advanced tumors[3]. Complete resection is believed to be the only curative treatment; however, the reported rate of resectable disease at diagnosis is only 30%–40%, which has not improved with time[4]. The challenging anatomical structures around the pancreas and the invasion of the nearby vasculature have led to special considerations for surgical treatment. Biological resectability, as crucial as anatomical resectability, has been well-recognized recently. Clinicians frequently observed subtle or undetectable distant metastases progressed during treatment. Thus, downstaging or conversion chemotherapy plays a significant role in increasing the resectability in patients with pancreatic cancer[4].

HIGHLIGHTS

- A decade-long before and after study showed significant survival improvements in patients with pancreatic cancer treated with evolving multimodal strategies.
- Neoadjuvant chemotherapy and extended pancreatectomy are increasingly used and have contributed to prolonged overall and progression-free survival, with the paradigm shift achieving 49% reduction in mortality risk [hazard ratio (HR) 0.51] and 37% reduction in progression risk (HR 0.63).
- Lower CA19-9 levels, earlier pathological stage, adjuvant chemotherapy, and treatment in the later period were independently associated with better long-term prognosis.

Over the past decade, there has been a paradigm shift toward aggressive multimodal treatment incorporating neoadjuvant chemotherapy (NAC), advanced surgical strategies, including extended pancreatectomy (EP), and adjuvant therapies for patients diagnosed with pancreatic cancer^[^5,6^]^. The increasing utility of NAC increases resectability and improves the survival of patients with initially resectable diseases[7]. EP for borderline resectable and locally advanced pancreatic cancer yields a more considerable surgical margin, which improves R0 resection and tumor clearance[8].

This study evaluated the real-world impact of these evolving strategies over 10 years at a high-volume tertiary referral center. By comparing outcomes across the two treatment eras, we sought to quantify survival gains and identify factors associated with improved prognosis in pancreatic cancer. The study is reported according to STROCSS 2025 guidelines[9].

## Materials and methods

### Study design and population

A before-and-after study was conducted at a tertiary referral medical center. All clinical data were collected prospectively as input into the institute’s local surgical database. Analysis was conducted by updating the nearest disease and survival status. This study adhered to the Declaration of Helsinki, and the institutional review board provided ethical approval on 1 April 2024. This study was registered retrospectively in ClinicalTrials.gov.

All patients who underwent curative surgery for pancreatic adenocarcinoma between 2011 and 2022 were included. Patients with other pancreatic cancer subtypes, including acinar cell carcinoma, mucinous carcinoma, undifferentiated carcinoma, and intraductal papillary mucinous neoplasm with associated adenocarcinoma, were excluded from the analysis. Patients who underwent palliative or R2 resection were excluded from the study.

### Treatment algorithms

The cohort was divided into period A (2011–2017, *n* = 154) and period B (2018–2022, *n* = 251) based on institutional protocol changes implemented in 2018 (Supplemental Digital Content Table S1, available at: http://links.lww.com/JS9/H12).

### Period A protocol

- NAC reserved for borderline resectable pancreatic cancer (BRPC), locally advanced pancreatic cancer (LAPC), or metastatic disease.
- Upfront surgery standard for resectable disease.
- EP selectively used.

### Period B enhanced protocol

- NAC expanded to resectable disease with high-risk features (CA19-9 ≥ 100 U/mL, positive lymph nodes, portal-SMV contact, and suspected visceral invasion).
- Systematic conversion surgery protocols.
- Proactive EP with vascular reconstruction.
- Standardized multidisciplinary team decisions.

The treatment evolution was facilitated by progressive availability of chemotherapy agents through Taiwan’s National Health Insurance system, including S-1 (approved 2014), nab-paclitaxel (approved 2019), and FOLFIRINOX components (approved 2021), alongside institutional participation in landmark trials that validated these regimens in Asian populations (Supplemental Digital Content Table S2, available at: http://links.lww.com/JS9/H12). Detailed treatment protocols, chemotherapy regimens (Supplemental Digital Content Table S3, available at: http://links.lww.com/JS9/H12), and surgical techniques are provided in Supplemental Digital Content Methods S1–S2, available at: http://links.lww.com/JS9/H12.

### Definitions and classifications

Resectability was defined according to NCCN Clinical Practice Guidelines based on vascular involvement patterns. EP was defined per International Study Group on Pancreatic Surgery (ISGPS) criteria[10] including vascular resection and/or multi-visceral resection. Textbook outcomes were defined as the absence of mortality, major morbidity (Clavien-Dindo ≥III), clinically relevant pancreatic fistula/bile leak (Grade B/C), and 30-day readmission[11].

Arterial divestment was not classified as arterial resection, as it represents the removal of the tumor from the arterial adventitia without vascular reconstruction. This technique, which has become the preferred approach at our institution since 2021 for cases with arterial abutment, avoids the morbidity associated with arterial resection.

### Outcomes

Overall survival (OS) and progression-free survival (PFS) were the primary outcomes. OS was defined as the interval from the date of the initial diagnosis to the date of death from any cause. PFS was defined as the time from the initial diagnosis to the first radiologically documented disease recurrence (RECIST 1.1) or death from any cause, whichever occurred first. Secondary outcomes included surgical outcomes, complications (classified by Clavien-Dindo Classification and ISGPS criteria[12]), and Textbook outcome.

* Detailed treatment protocols, chemotherapy regimens, surgical techniques, outcome definitions, and surveillance are provided in Supplemental Digital Content Methods S1–S2, available at: http://links.lww.com/JS9/H12 and Supplemental Digital Content Table S1–S3, available at: http://links.lww.com/JS9/H12.

### Statistical analysis

Comparisons between groups were performed using chi-square tests or Fisher’s exact test for categorical variables, and Mann–Whitney *U* tests were used as appropriate. Statistical significance was set at *P* < 0.05.

Survival data were collected from patients’ electronic medical records, institutional cancer registries, and national death records to ensure a comprehensive capture of events. Survival analysis was performed using the Kaplan–Meier method, and differences between groups were assessed using the log-rank test. Hazard ratios (HRs) with 95% confidence intervals (CIs) were calculated using Cox proportional hazard models adjusted for relevant clinical and pathological variables. Optimal cut-point analysis for continuous variables (preoperative CA19-9) was performed using the cutpointr package in R, maximizing the Youden index for survival prediction. Multicollinearity was assessed using variance inflation factors; values <2.0 indicate no substantial collinearity.

Subgroup analyses were performed, stratified by resectability status (resectable, borderline resectable, locally advanced, and metastatic) and treatment approach (neoadjuvant therapy vs. upfront surgery). Factors with *P* value less than 0.2 in the univariate analysis were included in the multivariate analysis.

Sensitivity Analysis: To address potential selection bias, propensity score matching (PSM) was performed using baseline variables: age, sex, BMI, ASA score, ECOG status, Charlson Comorbidity Index, preoperative CA19-9, tumor location, clinical staging, resectability, and minimally invasive or open approaches. One-to-one nearest neighbor matching without replacement was performed using a 0.2 standard deviation caliper. Balance was assessed using standardized mean differences (SMD <0.2 considered well balanced). Matched cohort analysis used Cox models stratified by matched pairs. PSM was selected over inverse probability of treatment weighting (IPTW) because the before-and-after design produced non-overlapping patient profiles across periods, which would yield extreme IPTW weights and unstable estimates. PSM restricts analysis to the region of common support, providing stable estimates among comparable patients.

Statistical significance was set at *P* < 0.05. R version 4.4.1 (R Project for Statistical Computing, Vienna, Austria) was used for statistical analysis.

## Results

**Figure 1.**
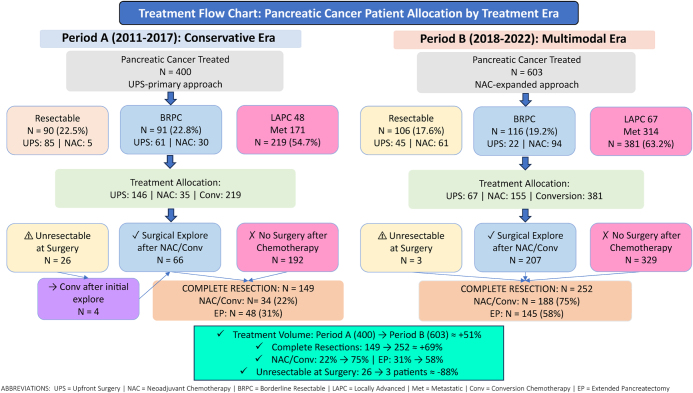
CONSORT flowchart showing patient selection process and treatment allocation.

Between 2011 and 2022, 1588 patients were diagnosed with pancreatic cancer at our institute. Among them, 1003 patients received anticancer treatment and follow-up (Fig. 1). The distribution by initial resectability status was resectable 19.2%, BRPC 21.1%, LAPC 11.1%, and metastatic disease 48.2%. NAC was administered to 34%, 60%, and 100% of the patients with resectable disease, BRPC, and LAPC/metastatic disease, respectively. The rates of surgical exploration were 96%, 79%, 41%, and 15%, and the complete resection rates were 89.8%, 62.8%, 33.9%, and 12.4%, respectively, for each subgroup.

## Trends in treatment strategies

**Figure 2.**
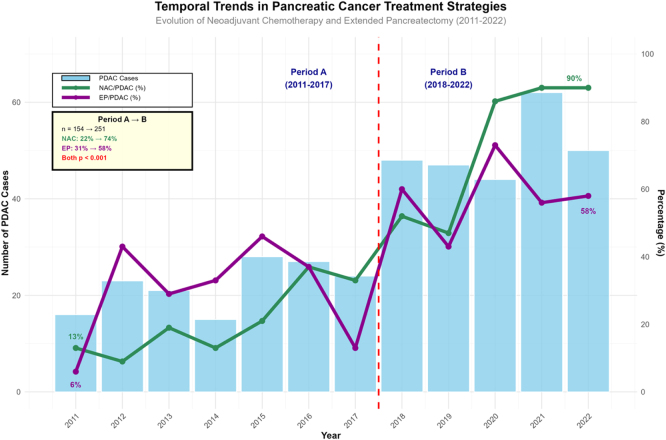
Temporal trends in treatment utilization from 2011 to 2022. Paradigm shift in pancreatic cancer management showing dramatic increase in neoadjuvant chemotherapy utilization from 6% to 90% (green line) and extended pancreatectomy from 13% to 58% (purple line).

**Figure 3.**
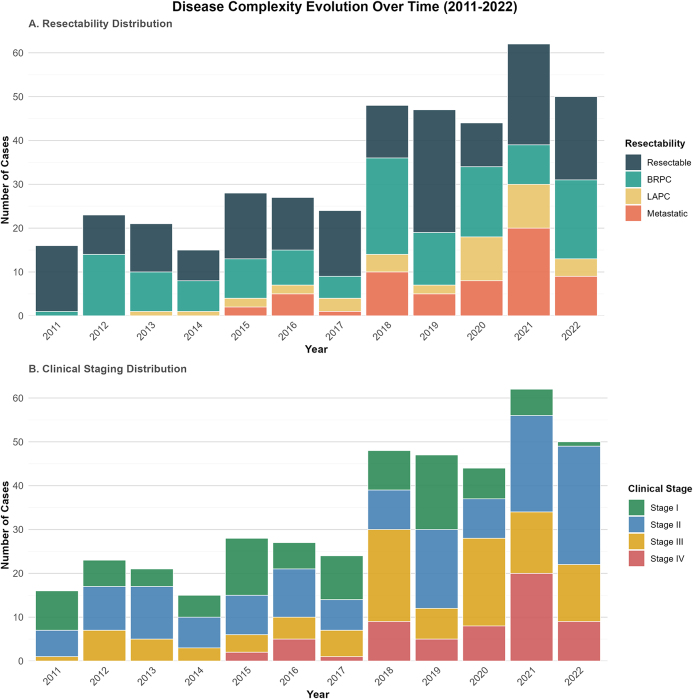
Disease complexity evolution over time. (A) Resectability distribution. Annual cases by resectability status: resectable (dark teal), borderline resectable (BRPC, teal), locally advanced (LAPC, yellow), and metastatic (coral). (B) Clinical stage distribution. Annual cases by clinical stage: Stage I (sea green), Stage II (steel blue), Stage III (goldenrod), and Stage IV (Indian red).

One thousand one hundred eighty-nine pancreatic resections were performed at our institution, with surgical experience in major pancreatic resections increasing from 60 to 120 cases annually. Among these, 405 patients were diagnosed with pancreatic adenocarcinoma and underwent curative resection: 154 during period A (2011–2017) and 251 during period B (2018–2022). There was a marked shift in treatment strategy between the two periods. NAC was administered to 74.9% of patients in period B compared to 22.1% in period A (*P* < 0.001), and EP increased from 31% to 58% (*P* < 0.001; Table 1). The annual trends showed a paradigm shift in treatment strategies, as (the number of cases and the ratio of NAC to EP showed a paradigm shift in treatment strategies; Fig. 2), with patients in period B presenting with more complex diseases (Fig. 3A) with advanced stages (Fig. 3B).

**Table 1:** The comparison of etiologies for surgery, severity of pancreatic cancer, treatment and surgical strategies, between period A (2011–2017) and period B (2018–2022).

	Period A*^1^*	Period B*^1^*	*P*-value*^2^*
Total numbers of major pancreas resection	***N* = 551**	***N* = 638**	
*Etiology*			<0.001
Benign etiology	205 (37.2)	176 (27.6)	
Pancreatic adenocarcinoma	154 (27.9)	251 (39.3)	
Other malignancy	192 (34.8)	211 (33.1)	
Pancreatic cancer	***N* = 154**	***N* = 251**	
*Treatment strategy*			<0.001
Neoadjuvant chemotherapy	34 (22.1)	188 (74.9)	
Upfront surgery	120 (77.9)	63 (25.1)	
*Surgical strategy*			<0.001
Standard pancreatectomy	106 (69)	106 (42)	
Extended pancreatectomy	48 (31)	145 (58)	
Multi-visceral resection	22 (46)	35 (24)	
Vascular resection	22 (46)	95 (66)	
Multi-visceral + vascular	4 (8)	15 (10)	

^1^ Median (Q1, Q3) or frequency (%).

^2^ Fisher’s exact test; Pearson’s Chi-squared test.

Resectability and clinical stage are defined according to NCCN Guidelines^®^, version 2.2025. BRPC, borderline resectable pancreatic cancer; LAPC, locally advanced pancreatic cancer.

## Surgical and perioperative outcomes

EP involved significantly longer operative time (326 vs. 265 min, *P* < 0.001), greater blood loss (450 vs. 200 ml, *P* < 0.001), and longer hospital stays (16 vs. 14 days, *P* < 0.001) compared to standard pancreatectomy (SP) (Table 2). EP resulted in higher major morbidity (20% vs. 11%, *P* = 0.019) and mortality rate (4.1% vs. 1.4% overall; 2.1% vs 0.5% 30-day mortality). Detailed mortality case analysis is provided in Supplemental Digital Content Table S4, available at: http://links.lww.com/JS9/H12.

**Table 2:** The comparison of demographics and surgical outcomes between the patients who received standard and extended pancreatectomy.

	Overall*^1^*	Standard Pancreatectomy*^1^*	Extended Pancreatectomy*^1^*	*P*- value*^2^*
	*N* = 405	*N* = 212	*N* = 193	
Sex				0.6
Female	190 (47)	97 (46)	93 (48)	
Male	215 (53)	115 (54)	100 (52)	
Age, year	64 (56, 71)	65 (57, 73)	63 (54, 69)	0.002
BMI, kg/m^2^	23.2(21.3, 25.7)	23.4 (21.6, 26.3)	23.0 (21.1, 25.3)	0.11
ASA				0.5
1–2	192 (47)	98 (46)	95 (49)	
3–4	213 (53)	114 (54)	98 (51)	
NAC	221 (55)	86 (41)	136 (70)	< 0.001
MIS	37 (9.1)	28 (13)	9 (4.7)	0.003
Optime, min	290 (233, 362)	265 (211, 319)	326 (260, 403)	< 0.001
EBL, ml	300 (150, 550)	200 (100, 400)	450 (250, 800)	< 0.001
ICU stay, day	0 (0, 3)	0 (0, 3)	2 (0, 4)	< 0.001
LOS, day	15 (12, 20)	14 (11, 18)	16 (12, 22)	< 0.001
PPH, Grade B/C	11 (2.7)	4 (1.9)	7 (3.6)	0.3
BL, Grade B/C	10 (2.5)	3 (1.4)	7 (3.6)	0.2
POPF, Grade B/C	30 (7.4)	11 (5.2)	19 (9.8)	0.074
Reoperation	15 (3.7)	5 (2.4)	10 (5.2)	0.13
Readmission	37 (9.1)	20 (9.4)	17 (8.8)	0.8
Overall Complications	199 (51)	113 (53)	93 (48)	0.2
Clavien-Dindo				
Classification				
Grade I	38 (9.4)	19 (9)	19 (9.8)	
Grade II	99 (24)	56 (26)	43 (22)	
Grade III	39 (9.7)	16 (7.6)	23 (11.6)	
Grade IV	12 (3)	5 (2.4)	7 (3.6)	
Grade V	11 (2.6)	3 (1.4)	8 (4.1)	
Major morbidity (CD grade ≥3)	62 (15)	24 (11)	38 (20)	0.019
30-day mortality	5 (1.2)	1 (0.5)	4 (2.1)	0.2
90-day mortality	8 (2)	2 (0.9)	6 (3.1)	0.2
Textbook Outcome	320 (79)	174 (82)	146 (76)	0.11

^1^ Median (Q1, Q3) or frequency (%).

2 Fisher’s exact test; Pearson’s Chi-squared test.

NAC, neoadjuvant chemotherapy; MIS, minimally invasive surgery; EBL, estimated blood loss; LOS, length of hospital stay (postoperative); PPH, post-pancreatectomy hemorrhage; BL, bile leak; POPF, postoperative pancreatic fistula.

Comparing between periods, EP mortality decreased markedly from 10.4% in period A to 2.1% in period B, and 30-day mortality improved from 6.3% to 0.7%. The annual distribution demonstrated improvement with accumulating experience (Supplemental Digital Content Figure S1, available at: http://links.lww.com/JS9/H12). Period B showed more frequent venous resections (70% vs. 52%, *P* = 0.027) but decreased resections of other organs, suggesting a more targeted approach (Table 3).

**Table 3:** The extension of surgical resections in extended pancreatectomy, comparing between the two periods.

	Overall*^1^*	Period A*^1^*	Period B*^1^*	*P*-value*^2^*
	*N* = 193	*N* = 48	*N* = 145	
Pancreas resection				0.2
PD/TP	125 (65)	27 (56)	98 (68)	
DP	68 (35)	21 (44)	47 (32)	
Arterial resection	16 (8.2)	2 (4.2)	14 (9.7)	0.4
SMA	3	0	3	
CA	9	1	8	
HA	4	1	3	
Venous resection	126 (65)	25 (52)	101 (70)	0.027
PV	12	3	9	
SMV	39	6	33	
PV-SMV junction	74	15	59	
IVC	1	1	0	
Type of venous resection				0.087
Tangential	13 (10)	3 (12)	10 (10)	
Segmental	113 (90)	22 (88)	91 (90)	
Stomach	16 (8.3)	8 (17)	8 (5.5)	0.029
Colon	33 (17)	16 (33)	17 (12)	<0.001
Adrenal	43 (22)	12 (25)	31 (21)	0.6
Kidney	6 (3.1)	2 (4.2)	4 (2.8)	0.6
Small bowel	6 (3.1)	2 (4.2)	4 (2.8)	0.6

^1^ Frequency (%).

^2^ Fisher’s exact test.

PD, pancreaticoduodenectomy; TP, total pancreatectomy; DP, distal pancreatectomy; SMA, superior mesenteric artery; CA, celiac axis; HA, hepatic artery; PV, portal vein; SMV, superior mesenteric vein; IVC, inferior vena cava.

## Survival outcomes

**Figure 4.**
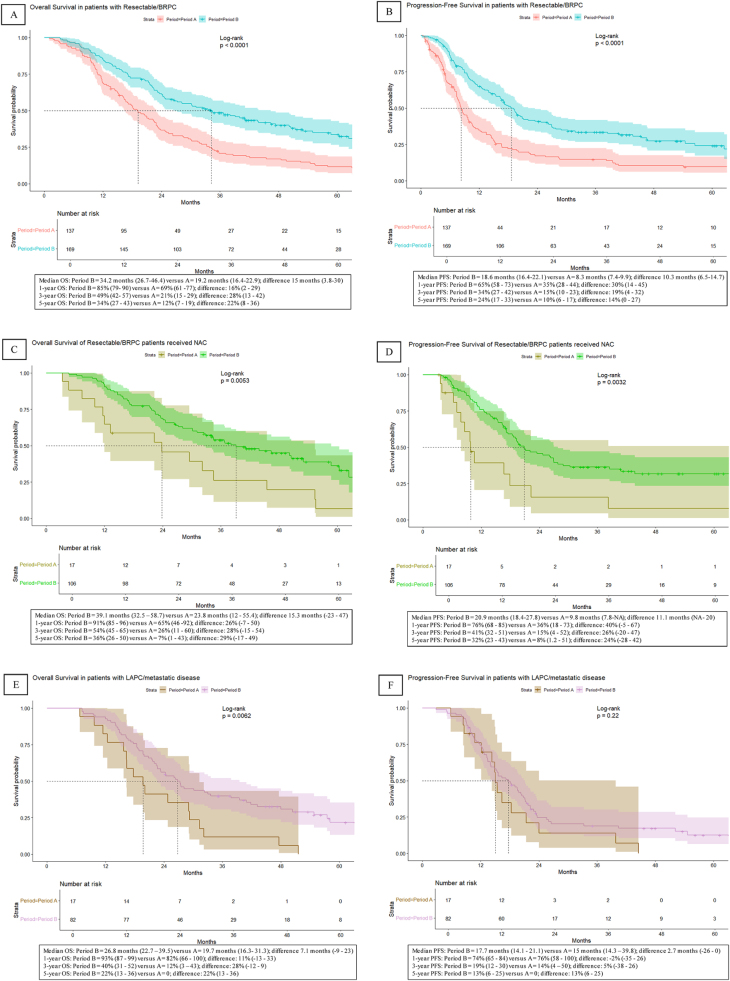
Kaplan-Meier curves of survival in the two periods (period A 2011–2017 and period B 2018–2022), stratified by resectability: OS (A) and PFS (B) for resectable/BRPC; OS (C) and PFS (D) for patients with resectable/BRPC received neoadjuvant chemotherapy; OS (E) and PFS (F) for LAPC/metastasis OS, overall survival; PFS, progression-free survival.

Median OS significantly improved from period A to period B. For resectable/BRPC, median OS increased from 19.2 to 35.5 months (*P* < 0.001; Fig. 4A), and PFS improved from 8.3 to 18.6 months (*P* < 0.001; Fig. 4B). In the subset of patients with resectable/BRPC disease who received NAC, patients in period B had a notably better survival curve than those treated earlier (OS: 39.1 vs. 23.8 months, *P* = 0.005; PFS: 20.9 vs. 9.8 months, *P* = 0.003; Fig. 4C,D). Among patients with LAPC and metastatic disease, median OS improved from 12.3 to 19.7 months (*P* < 0.001; Fig. 4E); meanwhile, PFS did not differ statistically (Fig. 4F).

Cox regression analysis demonstrated that period B was an independent predictor of improved outcomes (OS HR 0.51, *P* < 0.001; PFS HR 0.63, *P* < 0.001; Tables 4 and 5). This period effect remained robust even after adjusting for numerous clinicopathological variables, suggesting that broader institutional changes in treatment paradigms, such as increased NAC use and more targeted and aggressive surgical approaches, collectively contributed to improvements in survival.

**Table 4:** Cox regression models of risk factors for overall survival.

		Overall survival			
	Mortality	Univariate		Multivariate	
	*N* = 311	HR (95% CI)	*P* value	HR (95% CI)	*P* value
Age ≥ 65	159 (51)	1.27 (1.02–1.59)	0.036	1.28 (1.00–1.63)	0.045
Male	173 (56)	1.26 (1.01–1.57)	0.045	1.16 (0.92–1.47)	0.201
Tumor ≥ 4 cm	92 (30)	1.32 (1.03–1.68)	0.027	1.09 (0.84–1.42)	0.529
Resectability (ref. Resectable)					
BRPC	104 (33)	1.27 (0.98–1.65)	0.068		
LAPC	29 (9.3)	1.04 (0.70–1.56)	0.839		
Conversion	48 (15)	1.16 (0.83–1.61)	0.393		
Clinical stage (ref. Stage I)					
II	114 (37)	1.36 (1.01–1.84)	0.045	1.46 (1.03–2.07)	0.036
III	79 (25)	1.14 (0.82–1.57)	0.444	1.31 (0.84–2.05)	0.226
IV	47 (15)	1.25 (0.86–1.81)	0.245	1.26 (0.68–2.31)	0.465
Preoperative CA199 ≥ 70 U/ml	177 (57)	1.85 (1.48–2.32)	<0.001	1.54 (1.20–1.97)	<0.001
NAC	153 (49)	0.66 (0.53–0.83)	<0.001	1.00 (0.68–1.47)	0.800
Period B vs. period A	168 (54)	0.53 (0.43–0.67)	<0.001	0.51 (0.39–0.68)	<0.001
Extended pancreatectomy	153 (49)	1.16 (0.93–1.46)	0.180		
Vascular resection/divestment	108 (35)	0.96 (0.760–1.22)	0.741		
Achieved textbook outcome of surgery	237 (76)	0.66 (0.51–0.85)	0.002	0.75 (0.57–0.99)	0.040
Margin involved	108 (35)	1.45 (1.15–1.84)	0.002	1.18 (0.90–1.53)	0.225
Pathological stage (ref. Stage I)					
II	122 (39)	1.65 (1.24–2.20)	<0.001	1.29 (0.93–1.77)	0.124
III	73 (23)	2.88 (2.08–3.99)	<0.001	2.09 (1.42–3.08)	<0.001
IV	40 (13)	2.49 (1.69–3.66)	<0.001	2.34 (1.33–4.09)	0.003
Poor differentiation	60 (19)	1.45 (1.09–1.92)	0.010	1.29 (0.96–1.73)	0.087
Lymphovascular invasion	164 (53)	1.57 (1.26–1.96)	<0.001	1.10 (0.85–1.44)	0.472
Perineural invasion	275 (88)	1.82 (1.29–2.58)	<0.001	1.18 (0.81–1.73)	0.381
Adjuvant therapy	227 (73)	0.56 (0.44–0.72)	<0.001	0.56 (0.42–0.73)	<0.001

**Table 5:** Cox regression models of risk factors for progression-free survival.

		Progression-free survival			
	Progression	Univariate		Multivariate	
	*N* = 315	HR (95% CI)	*P* value	HR (95% CI)	*P* value
Age ≥ 65	148 (47)	0.89 (0.71–1.11)	0.303		
Male	167 (53)	1.13 (0.90–1.414)	0.291		
Tumor ≥ 4 cm	90 (29)	1.32 (1.03–1.69)	0.026	1.03 (0.79–1.35)	0.809
Resectability (ref. Resectable)					
BRPC	106 (34)	1.56 (1.20–2.02)	< 0.001	1.76 (1.260–2.48)	< 0.001
LAPC	31 (9.8)	1.32 (0.89–1.96)	0.171	1.81 (1.04–3.17)	0.037
Conversion	51 (16)	1.74 (1.25–2.41)	<0.001	3.27 (0.42–25.45)	0.258
Clinical stage (ref. Stage I)					
II	114 (36)	1.40 (1.43–1.88)	0.030	1.08 (0.77–1.51)	0.671
III	82 (26)	1.37 (0.99–1.89)	0.055	0.81 (0.51–1.28)	0.359
IV	50 (16)	1.81 (1.25–2.61)	0.002	0.62 (0.08–5.07)	0.657
Preoperative CA199 ≥ 70 U/ml	174 (55)	1.79 (1.43–2.24)	<0.001	1.62 (1.29–2.05)	<0.001
NAC	163 (52)	0.81 (0.65–1.01)	0.060		
Period B vs. period A	184 (58)	0.65 (0.52–0.81)	<0.001	0.63 (0.49–0.82)	<0.001
Extended pancreatectomy	153 (49)	1.25 (1.00–1.56)	0.049	1.04 (0.80–1.35)	0.788
Vascular resection/divestment	113 (36)	1.13 (0.90–1.42)	0.301		
Achieved textbook outcome of surgery	251 (80)	0.83 (0.63–1.09)	0.170		
Margin involved	106 (34)	1.65 (1.30–2.08)	<0.001	1.26 (0.97–1.64)	0.080<
Pathological stage (ref. Stage I)					
II	127 (40)	1.73 (1.31–2.28)	<0.001	1.19 (0.88–1.61)	0.267
III	67 (21)	2.59 (1.86–3.59)	<0.001	1.41 (0.97–2.06)	0.073
IV	39 (12)	2.89 (1.97–4.26)	<0.001	1.65 (0.98–2.78)	0.060
Poor differentiation	58 (18)	1.48 (1.11–1.97)	0.00	1.31 (0.97–1.77)	0.074
Lymphovascular invasion	167 (53)	1.73 (1.38–2.16)	<0.001	1.44 (1.10–1.88)	0.008
Perineural invasion	275 (87)	1.73 (1.24–2.42)	0.001	1.25 (0.87–1.80)	0.226
Adjuvant therapy	250 (79)	0.96 (0.73–1.27)	0.784		

For OS, additional significant negative prognostic factors included advanced age (≥65 years, HR 1.293, *P* = 0.045), elevated preoperative CA19-9 (≥70 U/ml, HR 1.498, *P* < 0.001), and advanced pathological stage (Stage III HR 2.09, *P* < 0.001; Stage IV HR 2.34, *P* = 0.003). Protective factors beyond period B treatment included achieving textbook surgical outcomes (HR, 0.75, *P* = 0.040) and receiving adjuvant therapy (HR, 0.56; *P* < 0.001; Table 4). PFS analysis revealed a somewhat different pattern, with tumor resectability (BRPC vs resectable, HR 1.76, *P* < 0.001; LAPC vs resectable, HR 1.81, *P* = 0.038) and lymphovascular invasion (HR 1.44, *P* = 0.008) emerging as essential determinants, whereas pathological staging and adjuvant therapy showed no significant impact on progression timing (Table 5). Wide confidence intervals observed for the conversion surgery category in the multivariate PFS analysis reflect the small sample size of this highly selected subgroup and represent appropriate uncertainty quantification rather than model instability – effect estimates for the primary variables of interest yield narrow confidence intervals and consistent significance across analyses.

## Sensitivity analysis

After PSM, 152 patients from period A were matched to 147 patients from period B (*n* = 299 total). Excellent balance was achieved across all baseline characteristics (Supplemental Digital Content Figure S2A–B, available at: http://links.lww.com/JS9/H12), with SMDs <0.2 for all variables (Supplemental Digital Content Table S5, available at: http://links.lww.com/JS9/H12). In the matched cohort, significant treatment differences persisted: NAC use (22% vs 64%, *P* < 0.001) and EP (31% vs 48%, *P* = 0.015), confirming that the matching preserved the key treatment interventions while controlling for baseline differences. Period B demonstrated superior survival outcomes with median OS improving from 19.2 to 30.3 months for resectable/BRPC (*P* < 0.001) and PFS from 8.3 to 14.3 months (*P* < 0.001; Supplemental Digital Content Figure S3A–B, available at: http://links.lww.com/JS9/H12). The period effect remained a strong predictor in multivariate analysis (OS HR 0.52, *P* < 0.001; PFS HR 0.60, *P* = 0.002).

## Discussion

This 10-year retrospective study reported the historical shift of treatment paradigm in a single medical center and the associated improvement in survival outcomes. The pre-and-post comparison highlights the evolution of multimodal strategies, including increased use of NAC, a higher frequency of extended resections, and a broader application of aggressive surgical principles. The impressive survival improvements seen from period B compared to period A showcase a significant advancement in our treatment approach. By the comprehensive strategy, we demonstrated a nearly 50% reduction in mortality risk (HR, 0.51) and a 37% reduction in progression risk (HR, 0.63), even after adjusting for numerous clinicopathological variables. These survival gains were most pronounced in patients with resectable or borderline resectable tumors; however, benefits were also observed in patients with locally advanced and metastatic disease^[^13,14^]^.

The expanded frequency of use of NAC from 22.1% to 74.9% represents the cornerstone of our institutional strategy, which is facilitated by progressive availability of chemotherapy agents through National Health Insurance. Despite of the reimbursement by NHI, the substantial delay compared to the landmark clinical trials worldwide limited the national application of NAC (Supplemental Digital Content Table S2, available at: http://links.lww.com/JS9/H12). The chemotherapy utilized in our institute is based on the real-world situation in Taiwan. Despite of the limited experience in standard regimens as mFOLFIRINOX, our data reported the great impact of NAC using regimens like SLOG or Gem/nab-Paclitaxel based regimen, which are more tolerated to Asian population. Our findings align with contemporary evidence supporting NAC accords all resectability status^[^15–19^]^. The expansion of NAC to includes resectable disease with high-risk features reflecting the growing recognition that anatomical resectability alone predicts surgical curability inadequately. The substantial improvement in PFS from 8.3 to 18.6 months for resectable/BRPC suggests more effect control of micrometastatic disease^[^20,21^]^. The benefits of NAC extended beyond tumor downstaging including improved patient selection for surgery. In our cohort, 15.3% of resectable and 31% of BRPC showed stable or progressive disease during neoadjuvant treatment, identifying patients unlikely to benefit from surgery. This selection effect, combined with biological downstaging, likely contributed to the superior outcomes observed in period B[22].

The increase in EP from 31% to 58% reflects our evolving surgical philosophy toward achieving optimal local control. While these complex procedures were associated with higher perioperative risks, including increased major morbidity (20% vs. 11%) and 30-day mortality (2.1% vs. 0.5%), they did not negatively affect long-term survival in multivariate analysis. This suggests that well-selected patients can benefit from aggressive surgery without compromising oncologic outcomes^[^23,24^]^. Moreover, our data revealed a shift toward more targeted extended resections over time, with increased vascular resections but decreased resections of adjacent organs such as the stomach and colon, potentially reflecting better patient selection and improved surgical techniques. These findings are consistent with those of other reports of extended pancreatectomies showing higher operative complexity and increased morbidity^[^25–27^]^.

The surgical mortality rate for EP (overall, 4.1% vs 1.4% for SP) requires careful contextualization with our institutional learning curve. Our detailed case analysis reveals that most deaths (7/11, 64%) occurred in conversion surgery cases representing the highest-risk patients with initially unresectable disease. The substantial improvement of the mortality rate over time, from 10.4% in period A to 2.1% in period B, reflects institutional learning curve completion and refined patient selection. This improvement paralleled our institutional growth from 60 to 120 annual major pancreatic resections. Contemporary literature supports the acceptability of our outcomes^[^27–29^]^. The achievement of textbook outcome emerged as an independent predictor of improved survival (HR 0.75, *P* = 0.040), highlighting the critical impact of surgical quality.

The pre-operative CA 19-9 served as an independent prognostic factor (OS HR 1.54, PFS HR 1.62, *P* < 0.001), reflecting the importance of biological resectability. CA 19-9, a sialyl Lewis A antigen, is expressed on pancreatic cancer cell surfaces. Its serum level directly correlated with tumor burden, biological aggressiveness, and metastatic potential^[^30–32^]^. Despite about 10% of the population (Lewis antigen-negative) cannot produce CA19-9^[^33^]^, the result from our study suggested its positive predictive value for high-risk patients, supporting its integration into treatment decision-making algorithms. Another independent significant predictor for OS in our analysis is adjuvant therapy (HR 0.56, *P* < 0.001). However, in multivariate analysis for PFS, adjuvant therapy showed no significant impact. The design of this study may not be able to provide thorough explanation due to high variation in treatment. Since the treatment choices were highly affected by the policy and reimbursement of medication by National Health Insurance in Taiwan, further investigation should be undertaken at national level (Supplemental Digital Content Table S2, available at: http://links.lww.com/JS9/H12)[34].

Our survival outcomes compared favorably with those reported in recent clinical trials on neoadjuvant treatment approaches^[^35,36^]^. The differences between the two periods highlight the importance of patient selection, surgical timing, and comprehensive perioperative care. The median survival of 35.5 months achieved in patients with resectable/BRPC in period B was comparable to the best outcomes reported in the literature for similarly selected patients^[^37–39^]^. The effect difference remained in the matched cohort. Survival analysis in our study also revealed a recurrence rate similar to that documented in other large cohorts^[^40,41^]^. The importance of achieving negative margins is a well-known survival predictor; however, it was not a significant factor in the multivariate analysis of this study, which was possibly associated with a wide range of resectability or staging in our patients. Nevertheless, tumor burden control and/or better tumor behavior are strong survival predictors, as our data support, including lower CA19-9 levels, less lymphovascular invasion, better tumor resectability, and lower clinical or pathological stages.

This single-center, before-after study design had inherent limitations. Temporal nature introduces potential confounding from simultaneous improvements including evolving surgical techniques, enhanced peri-operative care, and accumulated team experience. While PSM effectively controlled for measured baseline differences, unmeasured confounders may persist. PSM was employed rather than IPTW for sensitivity analysis, as the evolution in surgical indications between periods would generate extreme weights for patient profiles absent in period A. Future multi-center studies could employ IPTW or doubly robust methods to validate these findings further. The single-center design also limits generalizability, particularly to lower-volume centers without similar infrastructure. However, our standardized protocols and transparent reporting may serve as a model for other institutions implementing similar strategies. Selection bias remains inherent to the design, as treatment allocation was not randomized and protocols evolved systematically. Observational nature cannot replace randomized controlled trial evidence. Nevertheless, given the established efficacy of neoadjuvant therapy in BRPC/LAPC and still under-debate for resectable disease, our real-world evidence provides valuable insights into practical implementation of evolving treatment paradigms. Future studies with larger sample sizes should explore potential interaction effects between NAC and specific EP subtypes to optimize patient selection.

## Conclusion

Our study demonstrated that an aggressive multimodal approach incorporating the expanded use of NAC and extended surgical resections significantly improved outcomes in patients with pancreatic cancer. The paradigm shift observed at our institution aligns with the evolving international consensus on managing this challenging disease. Future studies are required to validate generalizable patient selection criteria, guide definitive downstaging therapies, and optimize perioperative care.

## Data Availability

The datasets used and/or analyzed in the current study are available from the corresponding author upon reasonable request.

## Research registration unique identifying number (UIN)

The study was registered retrospectively in the ClinicalTrials.gov, UIN: NCT06938906.
